# Phosphoproteins regulated by heat stress in rice leaves

**DOI:** 10.1186/1477-5956-9-37

**Published:** 2011-06-30

**Authors:** Xinhai Chen, Wenfeng Zhang, Baoqian Zhang, Jiechao Zhou, Yongfei Wang, Qiaobin Yang, Yuqin Ke, Huaqin He

**Affiliations:** 1 Key Laboratory of Ministry of Education for Genetic, Breeding and Multiple Utilization of Crops, Fuzhou 350002, China; 2 College of Life Sciences, Fujian Agriculture and Forestry University, Fuzhou 350002, China; 3 College of Horticulture, Fujian Agriculture and Forestry University, Fuzhou 350002, China

## Abstract

**Background:**

High temperature is a critical abiotic stress that reduces crop yield and quality. Rice (*Oryza sativa *L.) plants remodel their proteomes in response to high temperature stress. Moreover, phosphorylation is the most common form of protein post-translational modification (PTM). However, the differential expression of phosphoproteins induced by heat in rice remains unexplored.

**Methods:**

Phosphoprotein in the leaves of rice under heat stress were displayed using two-dimensional electrophoresis (2-DE) and Pro-Q Diamond dye. Differentially expressed phosphoproteins were identified by MALDI-TOF-TOF-MS/MS and confirmed by Western blotting.

**Results:**

Ten heat-phosphoproteins were identified from twelve protein spots, including ribulose bisphos-phate carboxylase large chain, 2-Cys peroxiredoxin BAS1, putative mRNA binding protein, Os01g0791600 protein, OSJNBa0076N16.12 protein, putative H(+)-transporting ATP synthase, ATP synthase subunit beta and three putative uncharacterized proteins. The identification of ATP synthase subunit beta was further validated by Western-blotting. Four phosphorylation site predictors were also used to predict the phosphorylation sites and the specific kinases for these 10 phosphoproteins.

**Conclusion:**

Heat stress induced the dephosphorylation of RuBisCo and the phosphorylation of ATP-β, which decreased the activities of RuBisCo and ATP synthase. The observed dephosphorylation of the mRNA binding protein and 2-Cys peroxiredoxin may be involved in the transduction of heat-stress signaling, but the functional importance of other phosphoproteins, such as H^+^-ATPase, remains unknown.

## Background

The inherent immobility of plants limits their abilities to avoid stress, requiring them to cope with abiotic stresses through innate defense mechanisms [1]. Some abiotic stresses, such as drought, salinity, extreme temperatures, chemical toxicity and oxidative stresses, are serious threats to agriculture and abiotic stresses have become the primary cause of crop loss worldwide, reducing average yields for most major crop plants by more than 50% [2]. High temperature is one of the most important abiotic stresses that reduce crop yield and quality [3,4]. Rice production is likely to be affected severely by an increase in mean global temperature [5,6]. The overall global temperature has steadily increased in recent decades due to rapid increases in atmospheric greenhouse gas concentrations [7,8]. The average global temperature has increased by 0.6°C in the 20th century and is expected to continue to rise 1.4-5.8°C by the end of this century [9,10]. Understanding the mechanisms by which rice respond to heat stress would facilitate the development of heat-tolerant cultivars with improved productivity in a warmer future climate.

Several physiological mechanism of rice plants for heat tolerance had been identified, including the selectively up-regulated of Rubisco activase large isoform, the increase of multiple enzymes of the Calvin Cycle, a fall in Ferredoxin-NADP(H) oxidoreductase (FNR) and a consistent increase in expression of a thiamine biosynthesis protein (THI1) [11]. Rice plants can remodel their proteomes in response to high temperature stress. Lee et al. [8] found that HSPs and energy- and metabolism-associated proteins were the major proteins affected by a high temperature of 42°C in leaves. In another study, lignification-related proteins were regulated by high temperature, and distinct proteins related to protection were up-regulated at different high temperatures, indicating that different strategies were adopted at different levels of high temperature: the higher the temperature, the greater the involvement of the protection machineries [6].

Given the importance of protein phosphorylation in the regulation of cellular signaling, a major goal of current proteomic efforts is the identification of phosphoproteins in higher organisms [12]. Phosphorylated proteins have been identified on a large scale in rice treated with various hormones [13], under high salinity stress [14] and under drought stress[12]. However, studies of protein phosphorylation induced by heat in rice have been limited.

Phosphoproteins are typically detected by one of three approaches. Proteins may be labeled with either inorganic phosphate (^32^Pi) *in vivo *or γ-(^32^P)-ATP *in vitr*o [15]. However, γ-(^32^P)-ATP-labeling *in vitro *may result in the false-positive identification of phosphoproteins, and (^32^P)-radiolabeling *in vivo *may induce radiation damage to cultured cells or tissues [16]. Phosphorylated amino acids can also be detected by antibodies specific for phosphotyrosine, phosphothreonine, or phosphoserine [17], but this approach is often time-consuming and costly. Another technology for detecting phosphoproteins is Pro-Q Diamond phosphoprotein gel stain, which allows direct, in-gel detection of phosphate groups attached to tyrosine, serine or threonine residues. Using Pro-Q Diamond dye, Jin et al. [18] observed 19 phosphorylated proteins in blood monocytes, and Chitteti and Peng [19] investigated dynamic changes in the phosphoproteome of *Arabidopsis cotyledon *during cell dedifferentiation.

In this study, we used two-dimensional gel electrophoresis (2-DE) followed by Pro-Q diamond dye and tandom MS to investigate differential regulation of rice phosphoproteins during heat stress.

## Results

### Pro-Q diamond staining of phosphoproteins

**Figure 1:**
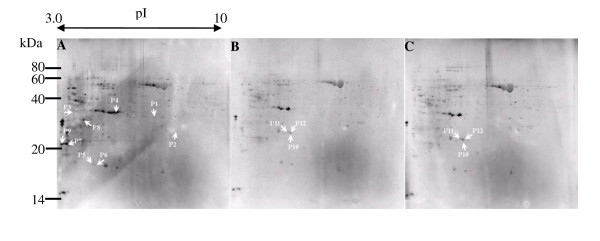
**2DE images of phosphoproteins extracted from leaves of rice under heat stress**. Proteins were separated by two-dimensional gel electrophoresis and stained by Pro-Q Diamond dye. We focused on the proteins that were phosphorylated or dephosphorylated in rice leaves under heat stress and labeled them P1, P2 and so on.  **A:** Phosphoproteins from the leaves of rice under normal conditions (Control).  **B: **Phosphoproteins from the leaves of rice under 12 h heat stress. ** C: **Phosphoproteins from the leaves of rice under 24 h heat stress.

Total proteins extracted from the leaves of rice in response to heat stress were separated by 2-DE and stained with Pro-Q Diamond dye. Overall 9 gel were analyzed, for each treatment in triplicates. More than 300 protein spots could be detected on each gel within a p*I *range of 3-10 and with relative molecular masses of 14-80 kDa (Figure 1). However, the majority of the visible phosphoproteins in the gel had a relatively low p*I *of 3-7 and high MW of 30-60 KDa (Additional file 1, Figure S1). Chitteti and Peng (2007) also found that rice root phosphoproteins stained with Pro-Q Diamond dye had low p*I *values. Furthermore, Pro-Q Diamond dye stain intensity is not proportional to protein concentration [14]. Therefore, in this study, we focused on the phosphorylated and dephosphorylated proteins and not the variability of phosphoproteins abundance in rice under heat stress.

### Specificity of Pro-Q diamond

To confirm the specificity of the Pro-Q Diamond for rice phosphoproteins, proteins extracted from the leaves of rice under heat stress were treated with CIP and then stained with Pro-Q or Coomassie blue dye (Additional file 2, Figure S2). Little staining by the Pro-Q Diamond dye was visible on the CIP-treated gel (Additional file 2, Figure S2), while protein staining by R-250 was not affected by CIP treatment (Additional file 2, Figure S2). These results indicate that Pro-Q Diamond specifically binds rice phosphoproteins, which is consistent with the findings of Chetteti and Peng [14]. Therefore, Pro-Q dye was used to detect rice phosphoproteins.

### Identification of heat-stress phosphoproteins

**Figure 2:**
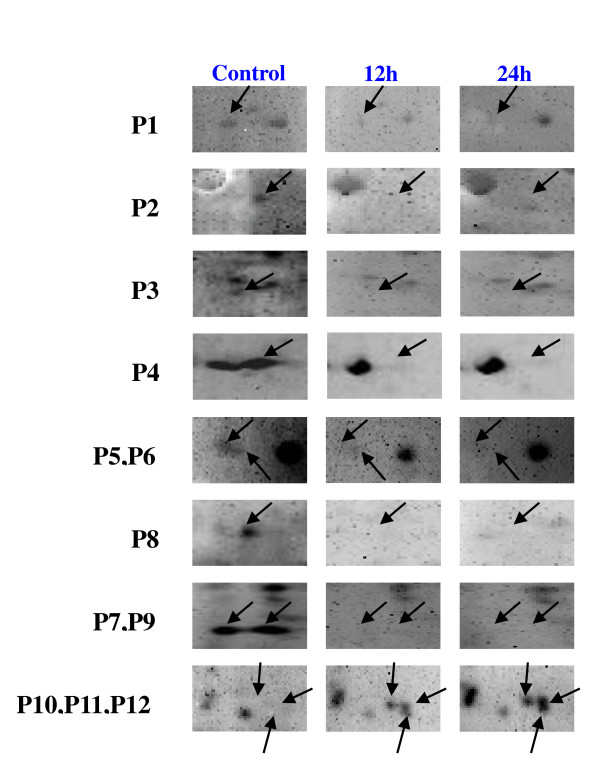
**The images of 12 protein spots, which were phosphorylated or dephosphorylated by heat stress**. Proteins, spots P1 to P9, were dephosphorylated by heat stress. However, proteins, spot P10, P11 and P12, were phosphorylated by heat stress.

Using the Pro-Q Diamond stain, we constructed rice phosphoprotein maps after 12 and 24 h of high temperature stress at 42°C (Figure 1). To investigate the response of the putative phosphoproteins to heat stress, we compared the phosphoprotein maps and analyzed the differentially phosphorylated proteins with ProteinMaster software 6.0 (FortuneSun Corporation, China). Twelve protein spots were identified as heat-responsive phosphoproteins in all three replicates, nine of which were dephosphorylated and three of which were phosphorylated in the leaves of rice after 12 h and 24 h of high temperature stress (Figure 2).

These differentially phosphorylated protein spots were manually excised, digested with trypsin and identified with an ABI 4700 Proteomics Analyzer. The twelve phosphoproteins were molecularly identified with high confidence (Table 1).

**Table 1:** Twelve phosphoproteins in leaves of rice were identified by MALDI-TOF-TOF-MS/MS.

**Spot No**.	**Accession no**.	Theoretical Mr (kDa)/p*I*	Observed Mr (kDa)	No. of Peptides	Coverage (%)	Putative Protein	Regulated by heat
P1	B8AXZ6	8.74/38.6	36.7	9	32	Putative uncharacterized protein	DP
P2	D3W4H3	7.00/26.5	25.4	7	27	Ribulose bisphosphate carboxylase large chain	DP
P3	Q6ER94	5.67/28.3	37.3	4	21	2-Cys peroxiredoxin BAS1, chloroplastic	DP
P4	Q8GTK8	7.68/41.2	38.1	8	26	mRNA binding protein	DP
P5	Q5K3B1	6.23/53.3	18.2	15	23	Ribulose bisphosphate carboxylase large chain	DP
P6	C7IWD0	4.92/41.9	18.1	10	26	Os01g0791600 protein	DP
P7	Q6ER94	5.67/28.3	22.5	5	30	2-Cys peroxiredoxin BAS1, chloroplastic	DP
P8	Q7X7H3	6.75/39.8	32.6	6	22	OSJNBa0076N16.12 protein	DP
P9	Q6Z8K7	4.98/26.2	22.4	4	25	H(+)-transporting ATP synthase	DP
P10	Q6ENG7	5.38/53.9	27.8	7	22	ATP synthase subunit beta, chloroplastic	P
P11	Q94GY1	5.64/22.8	28.7	8	35	Retrotransposon, centromere-specific	P
P12	A2YJU3	9.75/11.9	28.2	4	53	Putative uncharacterized protein	P

The protein spot numbers are given in Figure 1. Protein accession are from the Uniprot database http://www.uniprot.org/. DP: Dephosphorylated by heat stress; P: Phosphorylated by heat stress. The same below.

Ten of the differentially phosphorylated protein spots were identified as Rubisco large chain (spots P2, P5), 2-Cys peroxiredoxin BAS1 (spots P3, P7), putative mRNA binding protein (spot P4), Os01g0791600 protein (spot P6), OSJNBa0076N16.12 protein (spot P8), putative H(+)-transporting ATP synthase (spot P9), chloroplastic ATP synthase subunit beta (spot P10) and Retrotransposon (spot P11) (Table 1). The remaining two protein spots were identified as putative uncharacterized proteins (spots P1, P12). That different spots, such as P3 and P7, identified as the same protein might be induced by co- or post-translational modifications.

### Immunoblotting analysis of ATP synthase subunit beta

**Figure 3:**
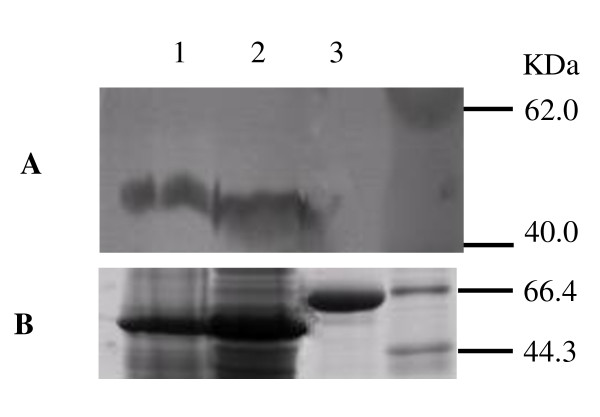
**The specificity of anti-ATP synthase subunit beta antibody**.CIP (a test protein) and total proteins extracted from rice and Arabidopsis were immunoblotted with anti-ATP-β antibody** (A)**, while parallel gels were stained with Coomassie blue **(B)**.  1. Total proteins extracted from rice leaves.  2. Total proteins extracted from Arabidopsis leaves.  3. CIP

The protein identification was verified by immunoblotting analysis, using ATP-β as a model protein. We first confirmed the specificity of the anti-ATP-β antibody. The total proteins extracted from rice and *Arabidopsis *were immunoblotted with the anti-ATP-β antibody; CIP was served as a negative control (Figure 3A). The parallel gels were stained with Coomassie blue (Figure 3B). *Arabidopsis *and rice proteins clearly reacted with the anti-ATP-β antibody, but CIP did not (Figure 3), confirming the specificity of the anti-ATP synthase β antibody.

**Figure 4:**
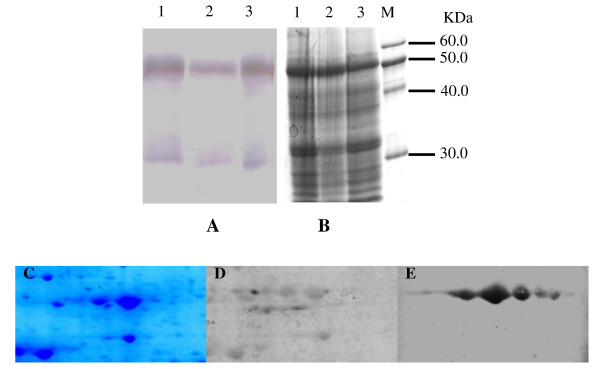
**The first-dimensional and second-dimensional immunoblotting analysis of ATP-β in leaves of rice under heat stress**. The proteins extracted from leaves of rice under heat stress were separated in 1D and 2-DE gels, transferred to an NC membrane, and then incubated with anti-ATP-β antibody.  **A:** Proteins on the 1D gel were probed with an anti-ATP-β antibody. ** B:** Proteins on the 1D gel were visualized by Coomassie blue staining.   **C:** Proteins on the 2-DE gel were visualized by Coomassie blue staining.  **D:** Proteins on the 2-DE gel were visualized by Pro-Q Diamond staining.  **E: **Proteins on the 2-DE gel were probed by anti-ATP-β antibody.  1: Proteins extracted from the leaves of rice under 24 h heat stress.  2: Proteins extracted from the leaves of rice under 12 h heat stress.  3: Proteins extracted from the leaves of rice under normal conditions (Control).  M: Marker.

The proteins extracted from rice leaves under heat stress were separated on 1D gels and 2-DE gels and then incubated with the anti-ATP-β antibody. Two immunoreactive bands were detected in 1D gels (Figure 4A), and five ptroteins exhibited strong immunoreactivity in 2-DE gels (Figure 4E). The rice ATP-β may have undergone co-or post-translational modifications that changed its p*I *and/or MW [20]. Alternatively, ATP-β may have diverse isoforms in the rice leaves. Riego et al. [21] have found six isoforms of ATP-β in barley and these six isoforms, which differed in their degrees of phosphorylation.

### Prediction of protein phosphorylation site

Phosphorylation site predictors are an important complement to the experimental characterization of protein phosphorylation. All 12 phosphosproteins detected by Pro-Q dye were also predicted to be phosphoproteins by prediction tools (Table 2). The two Rubisco large chains (spots P2 and P5) were predicted to be phosphorylated at Serine-221 and Serine-228 by Ataxia-telangiectasia mutated (ATM) kinase. Serine-32 and serine-35 of 2-Cys peroxiredoxin are potentially phosphorylated by Casein kinase I (CKI), cGMP-dependent protein kinase (PKG) and protein kinase C (PKC) delta. The putative mRNA binding protein regulated by heat could be a substrate of PKC and phosphorylated at threonine-59. Kinasephos predicted that spot P6 can be phosphorylated at Serine-165 by cell division control protein 2 (cdc2) and cyclin-dependent kinase (CDK). Serine-414 of ATP synthase subunit beta could be phosphorylated by casein kinase II or casein kinase I. The prediction tools also revealed that H^+^-ATPase may be phosphorylated at Serine-16 by the kinase of cdc2 and CDK (Table 2).

**Table 2:** The phosphorylation sites of 12 phosphoproteins were predicted by using different tools.

*Accession no.*	*Putative protein*	*Regulated* *by heat*	*Phosphorylation site *	*Tools*	Kinases
B8AXZ6	Putative uncharacterized protein	DP	Serine-33	Disphos Kinasephos NetphosK Scansite	cdc2, cdk5, ATM, MAPK
D3W4H3	RuBisCo large chain	DP	Serine-221	Kinasephos	ATM
Q5K3B1	RuBisCo large chain	DP	Serine-228	Kinasephos	ATM
Q6ER94	2-Cys peroxiredoxin BAS1, chloroplastic	DP	Serine-32	Disphos Kinasephos Scansite	CKI 14-3-3 Mode1
Q6ER94	2-Cys peroxiredoxin BAS1, chloroplastic	DP	Serine-35	Disphos Kinasephos Scansite	PKG PKC delta
Q8GTK8	Putative mRNA binding protein	DP	Threonine-59	Kinasephos NetphosK	PKC
C7IWD0	Os01g0791600 protein	DP	Serine-165	Kinasephos	cdc2, CDK
Q7X7H3	OSJNBa0076N16.12 protein	DP	Serine-18	Disphos Kinasephos Netphos2 Scansite	cdc2, MAPK cdc2 Casein kinase
Q7X7H3	OSJNBa0076N16.12 protein	DP	Serine-27	Disphos Kinasephos Netphos2 Scansite	Erk1 kinase
Q6Z8K7	Putative H(+)-transporting ATP synthase	DP	Serine-16	Disphos Kinasephos	cdc2, CDK
Q6ENG7	ATP synthase subunit beta, chloroplastic	P	Serine-414	Kinasephos Scansite	Casein kinase I, Casein kinase 2
Q94GY1	Retrotransposon, putative, centromere-specifi	P	Serine-127	Kinasephos Disphos	cdc2, ATM, IKK
A2YJU3	Putative uncharacterized protein	P	Serine-21	Disphos Kinasephos NetphosK	PKG, PKC

Phosphorylation site: The same phosphorylation site was predicted by different tools.

### Gene ontology (GO) analysis

Gene ontology analyses were performed for the 12 phosphoproteins to understand the biological processes and molecular functions involved in the rice heat stress response. Analysis of the biological processes revealed that three of the 12 variable phosphoproteins are involved in the Calvin cycle, two are part of hydrogen peroxide catabolism, two participate in ATP synthesis-coupled proton transport, one is involved in microtubule-based movement, and one was involved in cellular metabolic processes while the others are unknown (Table 3).

**Table 3:** Functional categorization of 12 phosphoproteins in leaves of rice under heat stress.

**Spot no**.	Putative Protein	Cellular component	Biological process	Molecular function
P1	Putative uncharacterized protein	NA	NA	transferase activity, transferring glycosyl groups
P2	Ribulose bisphosphate carboxylase large chain	plastid Chloroplast	Calvin cycle, Carbon dioxide fixation, Photosynthesis	ribulose-bisphosphate carboxylase activity, magnesium ion binding
P3	2-Cys peroxiredoxin BAS1, chloroplastic	Chloroplast, plastid	cell redox homeostasis, hydrogen peroxide catabolic process	Antioxidant Oxidoreductase Peroxidase
P4	Putative mRNA binding protein	NA	Cellular metabolic process	catalytic activity, coenzyme binding
P5	Ribulose bisphosphate carboxylase large chain	Chloroplast	Calvin cycle, Photosynthesis	ribulose-bisphosphate carboxylase activity, monooxygenase activity
P6	Os01g0791600 protein	photosystem	carbon utilization by fixation of carbon dioxide, photosynthetic electron transport chain	ribulose-bisphosphate carboxylase activity, NADH dehydrogenase (ubiquinone) activity
P7	2-Cys peroxiredoxin BAS1, chloroplastic	Chloroplast, plastid	cell redox homeostasis, hydrogen peroxide catabolic process	Antioxidant Oxidoreductase Peroxidase
P8	OSJNBa0076N16.12 protein	NA	NA	phosphatase activity
P9	Putative H(+)-transporting ATP synthase	plasma membrane	ATP synthesis coupled proton transport	hydrogen ion transporting ATP synthase activity, rotational mechanism
P10	ATP synthase subunit beta, chloroplastic	chloroplast thylakoid membrane, proton-transporting ATP synthase complex, catalytic core F(1)	plasma membrane ATP synthesis coupled proton transport	ATP binding, hydrogen ion transporting ATP synthase activity, rotational mechanism, hydrogen-exporting ATPase activity, phosphorylative mechanism, proton-transporting ATPase activity, rotational mechanism
P11	Retrotransposon, putative, centromere-specifi	NA	NA	NA
P12	Putative uncharacterized protein	NA	NA	NA

## Discussion

Twelve protein spots that exhibited altered phosphorylation in response to heat stress were identified by tandem mass spectrometry (Figure 1 and Table 1). RuBisCo is well known for its involvement in plant photorespiration (PR). *In vitro *phosphorylation of both the small and the large subunits of RuBisCo has been reported in spinach chloroplasts and rice [22,23]. Meanwhile, RuBisCo activity declines with dephosphorylation [22]. Thus, heat stress is likely to induce dephosphorylation of RuBisCo, resulting in the decrease of its activity.

Peroxiredoxins are a family of enzymes that catalyze the transfer of electrons from sulfhydryl residues to peroxides and are ubiquitously distributed among all organisms [24]. Peroxiredoxins have diverse functions in cellular defense-signaling pathways [25], and 2-Cys peroxiredoxin plays direct roles in cellular-signaling events [25-27]. Although its functional significance is unclear, heat-induced dephosphorylation of 2-Cys peroxiredoxin may be involved in the transduction of cellular-signaling.

Previous studies have provided strong *in vivo *and *in vitro *evidence that mRNA-binding proteins translocate to ribosomes and associate with mRNA to stabilize the mRNA, facilitating its translation or degradation [28,29]. Park et al. [30] found that the RNA encoding a small RNA-binding protein was involved in abiotic stress signaling. Moreover, ectopic over-expression of a glycine-rich RNA binding protein imparted high temperature stress tolerance to wild type yeast cells, suggesting that glycine-rich RNA binding protein probably binds and stabilizes stress-inducible transcripts under heat stress conditions [31]. mRNA binding proteins have been reported to be phosphorylated in the mammalian cell [32]. However, we previously found that the mRNA binding protein is an ABA-induced dephosphorylation protein [16], consistent with this study. Dephosphorylation of the mRNA binding protein may be involved in heat-stress signaling and may function to stabilize the heat-stress transcripts.

The phosphorylation of ATP-β in rice leaves under heat stress was confirmed by Pro-Q staining and western-blotting. The ATP-β is phosphorylated by casein kinase II [33]. The 14-3-3 proteins, which contain a casein kinase II phosphorylation motif [34], associated with the ATP synthases in a phosphorylation-dependent manner, remarkably depressing ATP synthases activity [35]. A reduction in ATP synthases activity has also been observed when rice leaves were exposed to heat stress [8]. Thus, Heat stress reduces ATP synthase activity in rice by phosphorylating ATP synthases.

However, another type of ATP synthase, putative H(+)-transporting ATP synthase (spot P9), was dephosphorylated in rice under heat stress. H(+)-transporting ATP synthase (H^+^-ATPase) in the plant plasma membrane acts as a primary transporter by pumping protons out of the cell, thereby creating pH and electrical potential differences across the plasmalemma [36]. The H^+^-ATPase is involved in salinity tolerance [37], but the functional importance of H^+^-ATPase dephosphorylation is unclear.

## Conclusion

In this study, ten differentially expressed phosphoproteins in rice leaves in response to heat stress were found. Heat stress induced the dephosphorylation of RuBisCo and the phosphorylation of ATP-β, decreasing the activities of RuBisCo and ATP synthase. The observed dephosphorylation of the mRNA binding protein and 2-Cys peroxiredoxin may be involved in the transduction of heat-stress signaling, but the functional importance of other phosphoproteins, such as H^+^-ATPase, remains unknown. Future work will validate or identify the functional importance of these heat-induced phosphoproteins.

## Methods

### Plant growth and heat stress treatment

Rice (*Oryza sativa *L. cv. Nipponbare) were germinated at 30°C for 48 h. The germinated seedlings were grown in a greenhouse at 28°C ± 2°C during the day and 22°C ± 2°C at night under natural light conditions (14 h light/10 h darkness period). Seedlings were grown in nutrient solution according to Li et al. [38]. For high temperature treatments, three-week-old rice seedlings were exposed to 42°C (Treatment) or 28°C (Control) for 12 h and 24 h [8]. Immediately after treatment, rice leaves were sampled and stored at -80°C.

### Two-Dimensional electrophoresis

Total protein extracts were prepared from fresh leaves according to He and Li [16] with some modifications. Briefly, frozen rice leaf tissue was finely ground in liquid nitrogen and suspended in icecold 10% w/v TCA in acetone containing 0.2% w/v DTT, incubated at -20°C overnight and centrifuged at 30,000 g for 25 min at 4°C. The pellets were collected and resuspended in 0.2% w/v DTT in acetone, incubated at -20°C for 4 h and centrifuged for 25 min at 30,000 g. This procedure was repeated three times and the pellets were lyophilized. The resulting samples were solubilized in lysis buffer (7 M urea, 2 M thiourea, 4% CHAPS, 2% Ampholine pH 3.5-10, 65 mM DTT and 0.5 mM PMSF) and then centrifuged at 30,000 g for 25 min at 4°C. The supernatants were collected and subjected to two-dimensional electrophoresis (2-DE). Protein concentrations were measured according to Bradford [39]. Focusing of the first dimension took place on a Protean IEF Cell (Bio-Rad, USA). After isoelectric focusing, the PH 3-10 IPG (17 cm, nonlinear) strips were immediately equilibrated in equilibration buffer (50 mM Tris-HCl, pH 8.8, 6 M urea, 30% glycerol, 2% SDS, 1% DTT and 0.002% bromophenol blue) and then placed directly onto 12% polyacrylamide-SDS slab gels. The gels were then stained by Pro-Q dye and Coomassie blue dye; parallel gels were probed by immunoblotting using a ATP-specific antibody.

### Detection of phosphoproteins

The 2-DE gels were stained with Pro-Q Diamond dye (Molecular Probes, USA) to detect phosphoproteins according to the manufacturer's instructions. 2-DE gels were fixed in solution (50% methanol, 10% acetic acid) and washed with distilled water three times. The gels were then stained with Pro-Q Diamond dye. After destaining (20% acetonitrile, 50 mM sodium acetate, pH 4.0), the gels were scanned with a TYPHOON TRIO+ scanner (GE Corporation, USA).

### Specificity of Pro-Q Diamond

To validate the specificity of Pro-Q Diamond for rice phosphoproteins, protein samples were prepared from rice leaves in extraction buffer (50 mM Tris-HCl pH 8.4, 100 mM NaCl, 1 mM MgCl_2 _and 1 mM DTT) excluding phosphatase inhibitors and incubated with Calf intestinal phosphatase (Promega Corporation, USA) or phosphatase buffer alone at 37°C for 2 h. The protein samples were then separated by SDS-PAGE, stained with Pro-Q Diamond or Coomassie blue dye, and scanned with a TYPHOON TRIO+ scanner.

### Analysis of 2-DE Gels

Images of 2-DE gels stained by Pro-Q dye and Coomassie blue dye were analyzed with ProteinMaster 6.0 software (FortuneSun Corporation, China). After spot detection and background subtraction (lowest on boundary mode), the 2-DE gels were aligned and matched, and the spot volumes were quantitatively determined (total spot volume normalization mode). The spots of interest were excised from the Coomassie blue dye stained gels with a scalpel and stored at -4°C in destaining solution (50% CAN, 100 mM NH_4_HCO_3_) for subsequent MS analysis.

### Mass spectrometry analysis and database searches

Protein spots were digested with sequencing-grade trypsin (Promega) as described previously [16]. The resulting peptides were desalted with C18 ZipTips (Millipore), mixed with 5 mg/ml alpha-cyanocinnamic acid in 70% acetonitrile and 0.1% trifluoroacetic acid, and spotted onto a MALDI sample plate. Mass spectra were acquired on a MALDI TOF/TOF mass spectrometer (4700 Proteomics Analyzer, Applied Biosystems) in both the MS and the MS/MS modes. Data were analyzed using MASCOT software (Matrix Science, UK). NCBI nr or Swiss-Prot was selected as the database. Typical search parameters were as follows: mass tolerance, 0.5 Da; missed cleavages, 2; enzyme, trypsin; fixed modifications, carbamidomethylation; variable modification, Oxidation (M); taxonomy, Oryza Sativa. For a match to be considered a valid identification, a confidence interval (C. I.) greater than 95% was required [12,16].

### Immunoblot analysis of ATP-β

Samples containing equivalent amount (30 μg) of protein were separated by SDS-PAGE on a 12.5% acrylamide gel (1-D). All samples were heated for 10 min in boiling water, cooled to room temperature and subjected to electrophoresis at a constant voltage of 100 V until the dye front reached the bottom of the gel. The gels were then probed by immunoblotting using ATP-β-specific antibody; parallel gels were stained with Coomassie blue stain.

The proteins in the 1D and 2-DE gels were transferred onto an NC membrane (Bio-Rad Corporation, USA) and immunoblotted with anti-ATP-β antibody as described previously [12,16]. Membranes were blocked with 5% (w/v) BSA in TBST (20 mM Tris-HCl, pH 7.5, 150 mM NaCl, 0.05% (v/v) Tween-20) for 1 hour at room temperature and then incubated with rabbit anti-ATP-β(AS05085, Agrisera) at a 1:1000 dilution for 3 h at room temperature. After being washed with TBST, membranes were incubated with goat anti-rabbit IgG conjugated with horseradish peroxidase at a 1:2,000 dilution for 1 hour. The membrane was then washed with TBST and developed with substrate (3,3'-diaminobenzidine) until optimum color developed.

### Prediction of phosphorylation site

Four recently published methods were selected to predict the phosphorylation site: Disphos [40], Kinasephos [41], NetphosK [42], Scansite [43]. We selected the *A. thaliana *option of Eukaryotes in Disphos for prediction. KinasePhos was used with 100% prediction specificity option, and no specific kinase was set. All the phosphoproteins were analyzed with the NetPhosK server without a filtering method, and a threshold of 0.7 was used to judge whether a site was predicted to be phosphorylated. Scansite was run by searching all motifs and the "high stringency level" setting was selected. Using these tools, we focused on obtaining the collective phosphorylation site(s) or distributive kinase(s), which is more reliable than using one method with one protein.

### Gene ontology (GO) annotation of phosphoproteins

Functional categorization of proteins was performed according to the GO rules using the gene ontology tools at http://www.agbase.msstate.edu. Three independent sets of ontologies were used to describe a gene product, the biological process, the molecular function and the cellular compartment [12].

## Abbreviations

HSPs: Heat shock proteins; ATP-β: ATP synthase subunit beta; RuBisCo: Ribulose bisphosphate carboxylase; CIP: Calf intestinal phosphatase; ATM: Ataxia-telangiectasia mutated kinase; CKI: Casein kinase I; PKG: cGMP-dependent protein kinase; PKC: protein kinase C; cdc2: cell division control protein 2; CDK: Cyclin-dependent kinase; NC: Nitrocellulose.

## Competing interests

The authors declare that they have no competing interests.

## Authors' contributions

HQH conceived of the study, designed experiments, analyzed data and wrote the manuscript. XHC designed and carried out experiments and wrote the manuscript, WFZ carried out the 2D-gels and wrote the manuscript. BQZ conducted the 1D gel experiment. JCZ performed the Pro-Q experiments. YFW analyzed protein data. QBY participated in the data analysis. YQK coordinated the study. All authors read and approved the final manuscript.

## Supplementary Material

Additional file 1 **Supplementary Figure 1**. 2-DE images of proteins extracted from leaves of rice under heat stress. Click here for file

Additional file 2 **Supplementary Figure 2**. The specificity of Pro-Q Diamond dye for rice phosphoproteins. Click here for file
