# Supplementary material for: Phosphoproteins regulated by heat stress in rice leaves
Source: Proteome Sci. 2011 Jun 30;9:37. doi: 10.1186/1477-5956-9-37 (PMC3150237; doi:10.1186/1477-5956-9-37)
Supplement: Additional file 1 — Supplementary Figure 1. 2-DE images of proteins extracted from leaves of rice under heat stress. [file 1477-5956-9-37-S1.DOC]

| Supplementary Figure 1. 2-DE images of proteins extracted from leaves of rice under heat stress. |
| --- |
| 10  P*I*  3.0  KDa  14  20  40  60  80 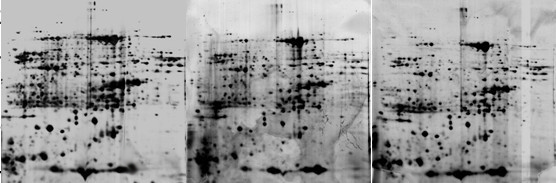 **A**  **B**  **C** |
| Proteins were separated by two-dimensional gel electrophoresis and stained with Coomassie blue dye.  A: 2-DE image of proteins from the leaves of rice under normal conditions (Control).  B: 2-DE image of proteins from the leaves of rice under 12 h heat stress.  C: 2-DE image of proteins from the leaves of rice under 24 h heat stress. |
