# Supplementary material for: Phosphoproteins regulated by heat stress in rice leaves
Source: Proteome Sci. 2011 Jun 30;9:37. doi: 10.1186/1477-5956-9-37 (PMC3150237; doi:10.1186/1477-5956-9-37)
Supplement: Additional file 2 — Supplementary Figure 2. The specificity of Pro-Q Diamond dye for rice phosphoproteins. [file 1477-5956-9-37-S2.DOC]

| Supplementary Figure 2. The specificity of Pro-Q Diamond dye for rice phosphoproteins. |
| --- |
| **R250**  **Pro-Q**  A B 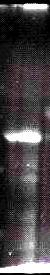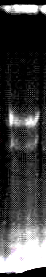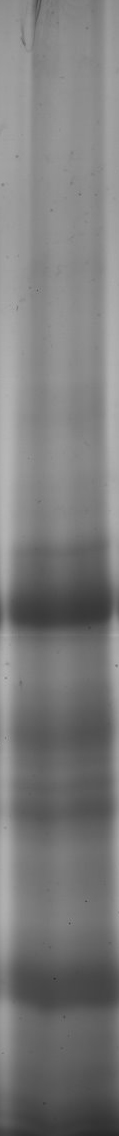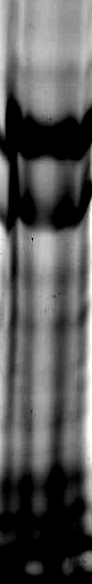 **CIP**  A B |
| Total proteins extracted from rice leaves were incubated with calf intestinal phosphatase (CIP) or phosphatase buffer alone at 37°C for 2 h, separated by SDS-PAGE, and stained with Pro-Q Diamond dye or Coomassie blue dye. The arrow indicates the CIP protein.  A: Pro-Q and Coomassie blue staining of proteins that were extracted from rice leaves and incubated with phosphatase buffer alone.  B: Pro-Q and Coomassie blue staining of proteins that were extracted from rice leaves and incubated with calf intestinal phosphatase. |
